# Prone positioning during veno-venous or veno-arterial extracorporeal membrane oxygenation: feasibility and complications after cardiothoracic surgery

**DOI:** 10.1186/s13054-022-03944-y

**Published:** 2022-03-21

**Authors:** Thibaut Genty, Quentin Cherel, Jacques Thès, Astrid Bouteau, Calypso Roman, François Stéphan

**Affiliations:** 1 Cardiothoracic Intensive Care Unit, Service de Réanimation adulte, Hôpital Marie Lannelongue, 133 Avenue de la Résistance, 92350 Le Plessis Robinson, France; 2 School of Medicine, Paris-Saclay University, Kremlin-Bicêtre, France; 3 INSERM U999, Pulmonary Hypertension: Pathophysiology and Novel Therapies, Hôpital Marie Lannelongue, Le Plessis-Robinson, France

Extracorporeal membrane oxygenation (ECMO) is a standard treatment for refractory hypoxaemia (veno-venous ECMO, VV-ECMO) and cardiogenic shock (veno-arterial ECMO, VA-ECMO). Severe hypoxaemia may persist despite ECMO. Prone positioning (PP) can improve outcomes of acute respiratory distress syndrome (ARDS) [1, 2]. However, few data exist on PP in hypoxaemic patients receiving VV-ECMO or VA-ECMO, particularly after cardiothoracic surgery. Here, we evaluated oxygenation and complications seen with PP during ECMO.

We retrospectively studied consecutive patients managed with PP and ECMO between August 2014 and December 2020. PP was used in patients with either refractory hypoxaemia (PaO_2_/FiO_2_ < 80 despite 100% FiO_2_ on ECMO) or persistent hypoxaemia (FiO_2_ requirement ≥ 80% with ECMO and lung condensations by CT). PP was chosen in patients on VA-ECMO because an additional venous cannula would have decreased arterial flow, potentially causing intolerance and, in the event of posterior basal pulmonary condensation, inducing adverse effects. We recorded ventilation and ECMO parameters, reason for PP, and complications. FiO_2 ECMO,_ FiO_2ventilator_, and PaO_2_ were collected before, during, and 6–12 h after PP.

Of 556 patients managed with ECMO, 34 (6.1%) (25 VV-ECMO, 9 VA-ECMO) received PP during ECMO (Table 1). PP significantly improved oxygenation (Fig. 1). Of the 87 PP sessions, six (6.9%) were followed by severe complications requiring emergent treatment. No patient experienced ECMO decannulation. Grade 3 or 4 pressure sores developed on the face or trunk in six (18%) patients. Of the 34 patients, nine (26%) died in the ICU. No patient died after ICU discharge. Of the 522 patients who received ECMO without PP, 237 (45.4%) died in the ICU, and median ECMO duration was 7 days [4–12].

**Table 1:** Characteristics and outcomes of the 34 patients managed with prone positioning during extracorporeal membrane oxygenation

Males/females, *n*	25/9
Age, years, mean ± SD	50.8 ± 16.3
BMI, kg/m^2^, mean ± SD	29.2 ± 6.3
SAPSII, mean ± SD	38.0 ± 11.8
*Reason for ICU admission, n*	
Thoracic surgery, *n* = 22	Pulmonary endarterectomy *n* = 11
	Lung transplantation, *n* = 7
	Lobectomy, *n* = 1
	Tracheal resection, *n* = 1
	ARDS after lung gunshot wound, *n* = 1
	Pleural/pulmonary abscess, *n* = 1
Heart surgery, *n* = 3	Heart transplantation, *n* = 1
	Bentall procedure, *n* = 1
	Aortic valve replacement, *n* = 1
Medical reasons, *n* = 9	ARDS due to COVID-19, *n* = 7
	Cardiogenic shock, *n* = 2
*Type of incision*^*a*^*, n*	
Sternotomy	15
Bi-thoracotomy	4
Clamshell incision	3
Thoracotomy	1
Thoracoscopy	1
Other	2
*Reason for ECMO*^*b*^	
VV-ECMO (*n* = 25)	Hypoxaemia due to ARDS/PGD, *n* = 25
VA-ECMO (*n* = 8) or VAV-ECMO (*n* = 1)	Cardiogenic shock, *n* = 5
	Residual PH, *n* = 4
*Complications during PP*^*c*^	
Circulatory arrest during an ECMO-VA membrane change	1
ECMO pump thrombosis related to HIT	1
Cardiac tamponade	1
Reperfusion-cannula displacement	1
Tracheostomy decannulation	1
Sternal wound infection	1
*PP session characteristics*^*d*^	
Number of PPs before ECMO implantation, median [IQR]	0 [0–1]
Number of PPs during ECMO, median/patient [IQR]	
All patients	2 [1–2.8]
VV-ECMO	2 [1–3]
VA-ECMO	2 [1, 2]
PP session duration, hours, mean ± SD	18.0 ± 4.2
Reason for PP	Refractory hypoxaemia *n* = 19
	Persistent hypoxaemia *n* = 15
*Ventilation parameters*^*e*^	
Patients with volume-controlled ventilation, *n* (%)	22 (65)
Patients with pressure-controlled ventilation, *n* (%)	12 (35)
Tidal volume, mL/kg predicted body weight, mean ± SD	4.5 ± 1.7
PEEP, cm H_2_O, median [IQR]	10 [10–15]
Respiratory rate, breaths/min, median [IQR]	20 [18–28]
*ECMO*^*f*^	
Blood flow, L/min, mean ± SD	
All patients	4.5 ± 1.7
VV-ECMO	4.6 ± 1.8
VA-ECMO	3.7 ± 1.1
Gas flow, L/min, mean ± SD	
All patients	5.6 ± 2.4
VV-ECMO	6.0 ± 2.4
VA-ECMO	3.9 ± 1.7
FiO_2 ECMO_, %, mean ± SD	
All patients	95 ± 12
VV-ECMO	95 ± 12
VA-ECMO	95 ± 13
Days on ECMO, median [IQR]	
All patients	15 [12–24]
VV-ECMO	15 [12–27]
VA-ECMO	15 [10–17]
Days from last PP to end of ECMO, median [IQR]	
All patients	5.5 [3.3–9.5]
VV-ECMO	5 [3–8]
VA-ECMO	8.5 [4.8–10.8]
Days from ECMO to first PP, median [IQR]	
All patients	7 [4–10]
VV-ECMO	6 [4–10]
VA-ECMO	7 [4–8]
Days from ICU admission to ECMO, median [IQR]	
All patients	2 [0–6.8]
VV-ECMO	3 [0–7]
VA-ECMO	0 [0–1]
*Outcome*	
ICU stay (days), median [IQR]	
All patients	31.5 [21–49]
VV-ECMO	32 [26–56]
VA-ECMO	19 [13–42]
Weaning off ECMO within 3 days after last PP, *n* (%)	
All patients	7/34 (21)
VV-ECMO	6/25 (24)
VA-ECMO	1/9 (11)
Death, *n* (%)	
All patients	9/34 (26)
VV-ECMO	5/25 (20)
VA-ECMO	4/9 (44)

*ECMO* extracorporeal membrane oxygenation, *FiO*_*2*_ fraction of inspired oxygen, *PEEP* positive end-expiratory pressure, *BMI* body mass index, *SAPS II* simplified acute physiology score version II, *ICU* intensive care unit, *ARDS* acute respiratory distress syndrome, *PGD* primary graft dysfunction, *PP* prone positioning, *PH* pulmonary hypertension, *HIT* heparin-induced thrombocytopenia, *IQR* interquartile range, *SD* standard deviation

^a^26 patients had a surgical incision. Among them, 25 underwent cardiothoracic surgery and one had a caesarean section. Patients managed with PP did not experience delayed wound healing or wound pressure sores. Subxiphoid drains but not laterothoracic drains were removed before PP sessions

^b^Among the five patients with cardiogenic shock, three had had heart surgery and two had shock due to medical reasons. The four patients with residual pulmonary hypertension had had pulmonary endarterectomy. No lung transplant recipients were on VA-ECMO at the time of PP

^c^One patient receiving peripheral VA-ECMO experienced a sternal infection, which was diagnosed before PP was started

^d^PP was performed according to a written standard procedure. All complications were reviewed after the session by the team. PP was expected to at least 16 h. However, the session could be shortened in the event of complications. At least seven staff members were required for turnings. An intensivist, a perfusionist, and a physiotherapist experienced in the management of PP were always among these seven staff members. One person focussed only on managing the head (intubation tube, central line, jugular cannula if any, nasogastric tube, and head support points) and another on managing the ECMO cannulas. The PP sessions were repeated according to the risk/ benefit ratio, i.e. to the balance between complications (mainly pressure sores) and improved oxygenation

^e^Maximum plateau pressure (cmH_2_O) was 30 cmH_2_O for both pressure-controlled and volume-controlled ventilation

^f^Patients receiving VA- or VV-ECMO were managed according to Extra-corporeal Life Support Organisation recommendations. ECMO was maintained until the respiratory and/or haemodynamic parameters improved. Weaning was conducted according to a local protocol. Briefly, VV-ECMO was explanted if the respiratory status did not deteriorate after 24 h of gas clamping. For VA-ECMO, a weaning test was performed with evaluation of haemodynamic and echocardiography parameters under 0.5 L/min of ECMO flow. Anticoagulation was with heparin to achieve an activated partial thromboplastin time equal to 1.5–2.0 times the control value. In the event of a bleeding complication, heparin was temporarily stopped. If the bleeding persisted, the ECMO oxygenator was changed

Atrio-septostomy was to be performed to unload the left ventricle if needed. However, none of our patients required this procedure

**Fig. 1:**
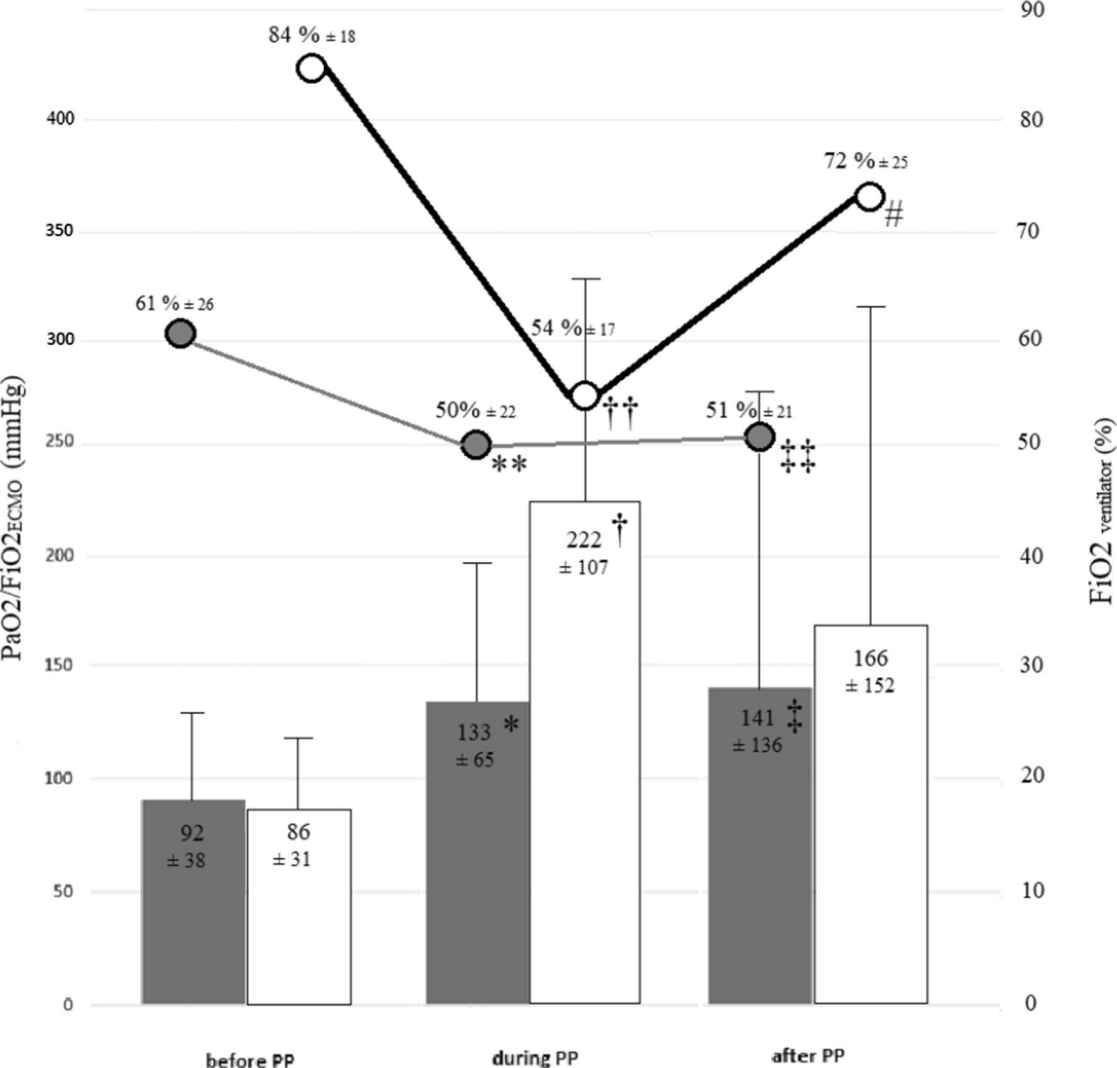
Oxygenation parameters before, during, and after prone positioning (PP) during veno-venous or veno-arterial extra-corporeal membrane oxygenation (ECMO). The grey bars and grey circles represent the PaO_2_/FiO_2 ECMO_ ratio and ventilator FiO_2_ values in patients receiving veno-venous ECMO. The open bars and open circles represent the PaO_2_/FiO_2 ECMO_ ratio and ventilator FiO_2_ values in patients receiving veno-arterial ECMO. Repeatedly measured quantitative variables were analysed by ANOVA. The PaO_2_/FiO_2 ECMO_ ratio changed significantly across time points in both the VA-ECMO group (*p* = 0.007) and the VV-ECMO group (*p* < 0.001). * VV-ECMO: PaO_2_/FiO_2 ECMO_ before PP/during PP, *p* = 0.007. ‡ VV-ECMO: PaO_2_/FiO_2ECMO_ before PP/after PP, *p* = 0.001. † VA-ECMO: PaO_2_/FiO_2ECMO_ before PP/during PP, *p* = 0.007. VA-ECMO: PaO2/FiO2_ECMO_ before PP/after PP, *p* = 0.148. ** VV-ECMO: FiO_2 ventilator_ before PP/during PP, *p* < 0.001. ‡‡ VV-ECMO: FiO_2 ventilator_ before PP/after PP, *p* < 0.001. †† VA-ECMO: FiO_2 ventilator_ before PP/during PP, *p* < 0.001. # VA-ECMO: FiO_2 ventilator_ during PP/after PP, *p* = 0.04

In patients receiving VV or VA-ECMO, PP improved oxygenation. Maintenance of the benefits after PP was most obvious in the VV-ECMO group. With VV-ECMO, the benefits of PP can be ascribed to well-documented mechanisms including a ventral-to-dorsal shift of tidal-volume distribution [2] and a decrease in the atelectasis very often seen after protective ventilation. With VA-ECMO, PP may be less likely to improve oxygenation, as gas exchange reflects the combined effect of VA-ECMO and of the native-lung ventilation/perfusion ratio, which is influenced by hypoxic vasoconstriction, shunting, alveolar collapse, and the dead space [3]. Hypoxaemia may worsen due to reduced pulmonary-artery flow during alveolar recruitment. We noted that the flow provided by the ECMO device remained constant during PP. As previously reported, cardiac output can increase, decrease or remain unchanged, depending on preload [4]. Finally, the beneficial effect of PP on the lung parenchyma outweighs the systemic hemodynamic effect even when cardiac output decreases.

In our study, ECMO duration before PP was 7 days, compared to 2 days in another study [2]. One quarter of our patients were successfully weaned off ECMO three days after the last PP session. Thus, PP may break the vicious circle of hypoxaemia, possibly allowing faster weaning off ECMO.

Another important result is the low frequency of complications, in keeping with earlier studies of VV-ECMO for ARDS [5, 6].

The main limitations are the retrospective design and single-centre recruitment of patients who underwent highly specific procedures such as lung transplantation or pulmonary endarterectomy.

Given the low frequency of severe complications, PP in patients under prolonged VA- or VV-ECMO may deserve consideration as a means of improving hypoxaemia and, perhaps, expediting weaning off ECMO.

## Data Availability

The dataset used and/or analysed during the current study is available from the corresponding author on reasonable request.
